# DNA methylation analysis of tumor suppressor genes in liquid biopsy components of early stage NSCLC: a promising tool for early detection

**DOI:** 10.1186/s13148-022-01283-x

**Published:** 2022-05-10

**Authors:** Α. Markou, D. Londra, V. Tserpeli, Ι. Kollias, E. Tsaroucha, I. Vamvakaris, K. Potaris, I. Pateras, Α. Kotsakis, V. Georgoulias, Ε. Lianidou

**Affiliations:** 1 Analysis of Circulating Tumor Cells (ACTC) Lab, Laboratory of Analytical Chemistry, Department of Chemistry, National and Kapodistrian University of Athens, Athens, Greece; 2 8th Department of Pulmonary Diseases, ‘Sotiria’ General Hospital for Chest Diseases, Athens, Greece; 3 Laboratory of Histology-Embryology, Molecular Carcinogenesis Group, Medical School, National and Kapodistrian University of Athens, Athens, Greece; 4 Department of Medical Oncology, University General Hospital of Larissa, Thessaly, Greece; 5 First Department of Medical Oncology, Metropolitan General Hospital of Athens, Cholargos, Greece

**Keywords:** CTCs, cfDNA, DNA methylation, NSCLC, Biomarkers, Liquid biopsy, Tumor suppressor genes

## Abstract

**Purpose:**

Circulating tumor cells (CTCs) and circulating tumor DNA (ctDNA) analysis represents a liquid biopsy approach for real-time monitoring of tumor evolution. DNA methylation is considered to be an early event in the process of cancer development and progression. The aim of the present study was to evaluate whether detection of DNA methylation of selected tumor suppressor genes in CTC and matched ctDNA provides prognostic information in early stage NSCLC.

**Experimental design:**

The methylation status of five selected gene promoters (*APC, RASSFIA1, FOXA1, SLFN11, SHOX2*) was examined by highly specific and sensitive real-time methylation specific PCR assays in: (a) a training group of 35 primary tumors and their corresponding adjacent non-cancerous tissues of early stage NSCLC patients, (b) a validation group of 22 primary tumor tissues (FFPEs) and 42 peripheral blood samples of early stage NSCLC patients. gDNA was isolated from FFPEs, CTCs (size-based enriched by Parsortix; Angle and plasma, and (c) a control group of healthy blood donors (*n* = 12).

**Results:**

All five gene promoters tested were highly methylated in the training group; methylation of *SHOX2* promoter in primary tumors was associated with unfavorable outcome. *RASSFIA* and *APC* were found methylated in plasma-cfDNA samples at 14.3% and 11.9%, respectively, whereas in the corresponding CTCs *SLFN11* and *APC* promoters were methylated in 7.1%. The incidence of relapses was higher in patients with a) promoter methylation of *APC* and *SLFN11* in plasma-cfDNA (*P* = 0.037 and *P* = 0.042 respectively) and b) at least one detected methylated gene promoter in CTC or plasma-cfDNA (*P* = 0.015).

**Conclusions:**

DNA methylation of these five gene promoters was significantly lower in CTCs and plasma-cfDNA than in the primary tumors. Combination of DNA methylation analysis in CTC and plasma-cfDNA was associated with worse DFI of NSCLC patients. Additional studies are required to validate our findings in a large cohort of early stage NSCLC patients.

**Supplementary Information:**

The online version contains supplementary material available at 10.1186/s13148-022-01283-x.

## Introduction

Lung cancer remains globally a leading cause of death from cancer, due to its high incidence and late-stage diagnosis. Annual imaging was recommended for screening high risk individuals, since early diagnosis of lung cancer at an early stage before it has been spread, could result to more successful treatments. However, less than 10% of those eligible adhere to guidelines [1] and about 45% of NSCLC patients even diagnosed at an early stage develop local or distant recurrence within 8–18 months. Moreover, 30% to 80% of patients with early stage NSCLC will die within 5 years of diagnosis [2].

Liquid biopsy (LB) analysis is nowadays a novel highly promising and very important tool in clinical oncology. LB is based on the analysis of several types of tumor-derived biomarkers that circulate in the bloodstream such as circulating tumor cells (CTCs), exosomes, and tumor-derived circulating nucleic acids, such as circulating tumor DNA (ctDNA), microRNAs (miRNAs) and non-coding RNAs (ncRNAs) [3, 4]. In 2005 the first LB test that was approved by the FDA was CTC enumeration in metastatic breast cancer based on the CellSearch® system [5] (Menarini, Silicon Biosystems, Italy) while in 2017 the first cfDNA-based liquid biopsy test was approved for EGFR mutations in plasma-cfDNA [6]. The number of FDA-approved tests that are based on the analysis of plasma-cfDNA in order to predict response and resistance to therapy is constantly growing [7, 8]. CancerSEEK and CancerEMC are newly developed liquid biopsy blood tests that have been proposed for early cancer detection based on a combination of circulating protein biomarkers and mutations in cfDNA from the blood [9, 10].

DNA methylation plays an essential role in regulating gene expression and modifying chromatin conformation [11]. Epigenetic biomarkers have been discovered in various types of cancer [12] since DNA methylation alterations are frequently observed and have diverse implications in carcinogenesis, diagnosis and prediction. Beyond DNA mutations, changes in DNA methylation patterns are known to arise early during cancer pathogenesis and in many cases hypermethylation in the promoter region of selected genes is thought to have carcinogenic and prognostic effects [13, 14]. A combination of DNA methylation analysis with LB is very powerful in identifying circulating epigenetic biomarkers of clinical importance [15]. Based on epigenetic alterations two commercially available tests for colorectal cancer and lung cancer detection in plasma-cfDNA levels namely “Epi proColon” (methylation in *SEPT*9) and “Epi proLung” (methylation in *PTGER4* and *SHOX2*), respectively, were used in clinical practice [16], [17]. Recently, a six-marker panel methylation-based plasma test, namely lung EpiCheck was validated for the detection of lung cancer [18].

Our group was the first to demonstrate epigenetic alterations in CTCs using as a model breast cancer [19, 20]. We have also reported the prognostic significance of DNA methylation markers in plasma-cfDNA of NSCLC patients [21, 22]. Several groups have also reported that promoter methylation of specific genes could be used as noninvasive circulating epigenetic biomarkers for the diagnosis of NSCLC [23–25].

In the present study the methylation status of five tumor suppressor genes, involved in some of the hallmarks of cancer [26], was investigated. The selection of the genes was based on a meta-analysis of RNA-seq data from The CancerGenome Atlas (TCGA) Consortium according to their limited expression in patients with NSCLC. The selected genes were: (a) *RASSF1A*, a tumor suppressor gene involved in cell proliferation and apoptosis [27] (b) *SHOX2* acting as transcription factor [28], (c) *SLFN11* that is described for its role in differentiation and cell proliferation [29], (d) *APC* that participates in cell migration, adhesion transcriptional activation and apoptosis [30] and (e) *FOXA1*, a transcription factor which positively regulates tumor growth and metastasis [31].

The aim of the present study was to evaluate the prognostic significance of DNA methylation of these five genes and to evaluate their prognostic significance in primary tumors, corresponding plasma-cfDNA and size-based enriched CTC fractions of patients with early stage NSCLC. Our data strongly suggest that combination of DNA methylation analysis in CTC and plasma-cfDNA provides prognostic significance in patients with early stage NSCLC.

## Materials and methods

### Clinical samples

Three different groups of clinical samples were analyzed:

- Training group: consisting of primary NSCLC (fresh-frozen) tissues and corresponding adjacent non-neoplastic tissues of 35 patients, all diagnosed with operable (stage I-III) NSCLC; tumor histology was squamous cell carcinoma (SCC; *n* = 15), adenocarcinoma (ADC; *n* = 13) and undifferentiated (NOS; *n* = 7). In this group the majority of patients (65.7%) were smokers. All patients were treatment naïve when the samples were collected but after surgery all patients received standard chemotherapy protocols for adjuvant NSCLC. A clinical relapse was documented in 19/35 (54.3%) patients during the follow-up period.
- Validation group: consisting of 42 patients with operable NSCLC (stage IA–IIIA). Plasma-cfDNA samples and the corresponding CTC were analyzed from all patients however corresponding primary FFPEs tumor tissues were available for analysis only for 22 patients. Peripheral blood from the patients was obtained before surgery and before the initiation of any systemic treatment; 14 patients were diagnosed with ADC, 24 with SCC and 4 with NOS NSCLC. In this group the majority of patients (78.6%) were smokers.
- Control group: consisting of plasma-cfDNA and matched CTC samples isolated from 12 healthy blood donors (HD). All HD had no known illness or fever at the time of blood drawing and were ≥ 35 years old.

All patients gave a written informed consent to participate in the study, which was approved by the Ethics and Scientific Committee of the Thoracic Diseases General Hospital “Sotiria”.

### Isolation of genomic DNA from fresh-frozen primary tumor tissues and formalin-fixed paraffin-embedded tissues

Genomic DNA (gDNA) from primary NSCLC fresh-frozen tissues and corresponding adjacent tissues was isolated using the DNeasy Blood and Tissue Kit (Qiagen, Hilden, Germany) according to the manufacturer’s instructions. DNA extraction of formalin-fixed paraffin-embedded tissues (FFPEs) was performed using QIAamp DNA FFPE Tissue Kit (Qiagen, Hilden, Germany). DNA concentration in all cases was measured with a NanoDrop 1000 Spectrophotometer (Thermo Scientific, USA).

### Isolation of plasma-cfDNA

Plasma was isolated from peripheral blood (in EDTA) within 2 h to 4 h from sample collection by centrifugation at 530xg for 10 min at room temperature. Once isolated, plasma samples were centrifuged again at 2.000xg for 10 min, before transferring into clean 2-mL tubes and freezing at − 70 °C until time of processing. cfDNA was extracted from 2.00 mL plasma using the QIAamp® Circulating Nucleic Acid kit 50 (Qiagen®, Germany), as previously described [32].

### CTCs enrichment using a size-based microfluidic device

The micro-fluidic device Parsortix (ANGLE plc, United Kingdom) [33] was used for the isolation of CTCs from 20 mL of peripheral blood in EDTA. A microscope slide sized disposable cassette was used for the division of blood components [34–36]. After this step, enriched CTCs were collected in a total volume of 200μL of PBS into Eppendorf tubes. The isolation of gDNA from enriched CTCs was performed using TRIZOL-LS (Thermo Fisher Scientific, United States) as previously described [19, 20].

### Sodium bisulfite conversion

Up to 500 ng cfDNA were chemically modified with sodium bisulfite (SB) as previously described [20, 22, 32, 34]***.*** In each SB-reaction negative and positive controls were included. The Universal Methylated Human DNA Standard (ZYMO Research) was used as fully methylated (100%) positive control. SB-converted DNA samples were stored at − 70 °C until further use.

### Whole bisulfite amplification

Whole genome amplification (WGA) of SB-converted DNA was performed using the EpiTect Whole-Bisulfitome Kit (Qiagen) as previously described [37]. This protocol is optimized for the amplification of > 50 ng of SB-converted DNA, diluted with nuclease-free water to a final volume of 10μL. The amplification was performed in a thermal cycler (Mastercycler® pro, Eppendorf) (28 °C/8 h, 95 °C/5 min, and 4 °C until storage; lid temperature set to 70 °C).

### Quality control

Positive and negative controls were included in all steps to ensure the quality and reproducibility of results. Human placental gDNA (Sigma-Aldrich) was used as a negative control after SB conversion. Universal Methylated Human DNA Standard (ZYMO Research) was used as fully methylated positive control. DNA integrity from all DNAs samples was checked by amplifying a region in exon 20 of the *PIK3CA* gene as previously described [38]. The quality of amplified SB-DNA was checked by a real-time PCR assay for β-actin (*ACTB*); only samples with positive amplification of *ACTB* were used for further analysis [39].

### Real-time MSP assays

DNA methylation of *RASSFIA*, *SLFN11* and *FOXA1* was detected by using sensitive and specific real-time MSP assays based on specific primers pairs for methylated promoter sequences as previously described [40]. For the detection of *APC* and *SHOX2* DNA methylation we designed in silico primers for using Primer Premier 5.00 software (Premier Biosoft) avoiding the formation of stable hairpin structures, primer dimers, cross dimers, and false priming sites. Optimization of experimental conditions including annealing temperature, time, concentrations of the primer pair, and finally buffer, MgCl_2_, dNTPs, and BSA concentrations (data not shown) was performed in order to develop sensitive and specific real-time MSP assays for *APC* and *SHOX2* promoter methylation.

One microliter of SB-converted DNA was added to 9 μL reaction mixture containing 0.05 U/μL − 1 GoTaq® Hot Start Polymerase (Promega, Maddison, WI, USA), 0.2 × of the supplied PCR buffer, 2 mM of MgCl_2_, 0.2 mM of each dNTP (Thermo Fisher Scientific), 0.3 μg/μLBSA, 0.3 μM of the forward and reverse primers, and 1 × LCGreen Plus Dye (Idaho Technology, Salt Lake City, Utah, USA). Finally, deionized water was added to a final volume of 10μL. Real-time MSP protocol began with one cycle at 95 °C for 2 min followed by 45 cycles of 95 °C for 10 s, 63 °C for 20 s, and 72 °C for 20 s. Immediately after amplification, a rapid cooling cycle to 40 °C for 30 s was introduced in order to prepare the melting curve acquisition step. Real-time fluorescence acquisition was set at the elongation step (72 °C). The following melting curve analysis included the steps of 55 °C for 10 s, 92 °C for 0 s with a ramp rate 0.11 °C/s (acquisition mode: continuous), 92 °C for 1 min, and 40 °C for 1 min.

### Statistical analysis

Correlations between methylation status and clinico-pathological features of the patients were assessed by using the Chi-square test. Disease-free interval (DFI) and overall survival (OS) curves were calculated by using the Kaplan–Meier method and comparisons were performed using the log-rank test. P-values < 0.05 were considered as statistically significant. Statistical analysis was performed by using the SPSS Windows version 17.0 (SPSS Inc., Chicago, IL, USA).

## Results

**Fig. 1:**
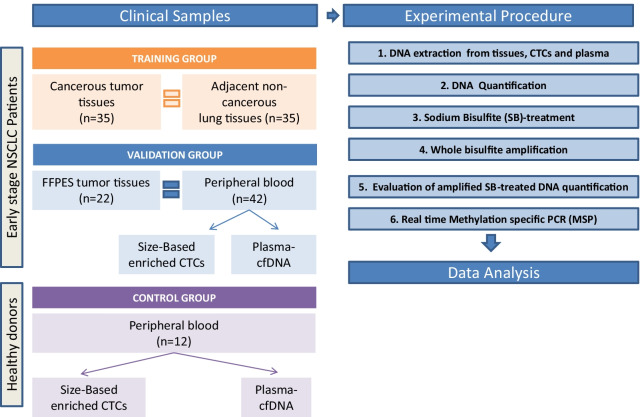
Outline of the experimental procedure

The outline of the study is shown in Fig. 1.

### DNA methylation analysis

#### Training group

**Fig. 2:**
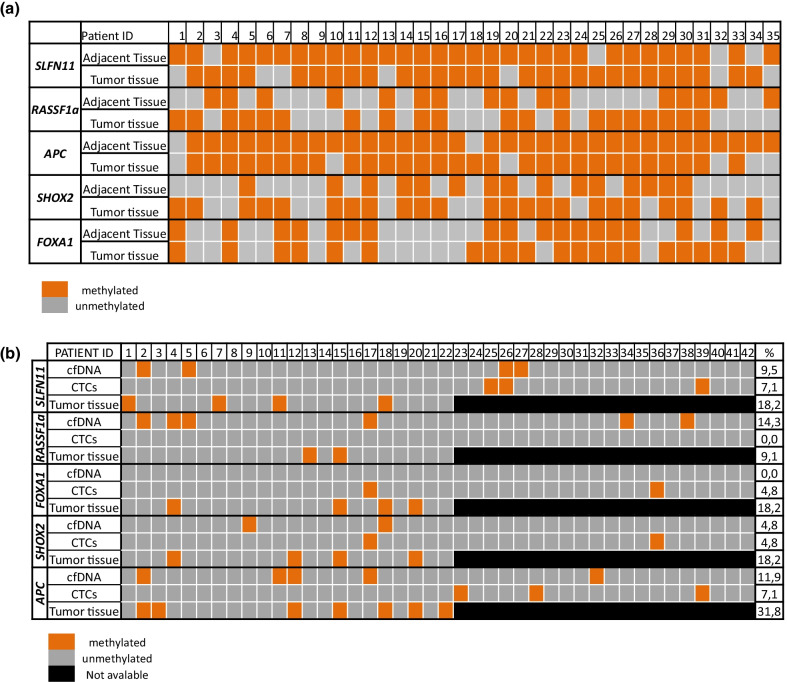
Methylation status of five selected genes in **a** a training group of NSCLC paired tissues (*n* = 35) and **b** in a validation group consisting of: 42 samples of size-based CTC fractions, 42 samples of corresponding plasma-cfDNA and 22 samples of corresponding FFPEs tumor tissues of early stage NSCLC patients

The methylation status of *APC, FOXA1, SHOX2, SLFN11* and *RASSFIA* gene promoters was first assessed in a training group of 35 pairs of primary NSCLC tissues and their adjacent non-cancerous tissues (Fig. 2a). In the primary fresh-frozen NSCLC tissue samples and the adjacent non-cancerous tissue, promoter DNA methylation was detected as follows: *APC* 82.8% and 85.7%, respectively*, FOXA1* 60% and 48.6%, respectively, *SHOX2* 65.7% and 42.8%, respectively, *SLFN11* 80% and 94.3%, respectively, and *RASSFIA* 60% and 45.7%, respectively. At least one gene was found methylated in all primary tumor tissues.

#### Validation group

Promoter methylation of these five genes was evaluated in an independent group of 42 patients with early NSCLC both in CTC and the corresponding plasma-cfDNA. For 22 of these patients, FFPEs from the primary tumor were also available.

##### DNA methylation analysis in primary tumors

Promoter methylation for at least one of these genes was detected in 12/22 (54.5%) of the primary tumor samples, in 2/22 (9.0%) of corresponding CTC and in 12/22 (54.5%) of corresponding plasma-cfDNA. *APC* was highly methylated in 7/22 (31.8%) of primary NSCLC tissues when compared to *SLFN11, SHOX2* and *FOXA1* (4/22: 18.2% each of them), whereas DNA methylation of *RASSF1a* was detected in only two samples (Fig. 2b).

In two patients (2/22, 9%), *APC* promoter methylation was detected both in the primary tumor and in the corresponding plasma-cfDNA. In 12 patients, at least one promoter of the tested genes was detected in the primary tumor but not in CTCs or plasma-cfDNA, whereas in six patients all tested gene promoters were negative for DNA methylation both in the primary tumor as well as in the in the size-based CTCs and plasma-cfDNA. In addition, in three patients promoter methylation was identified in the CTC or plasma-cfDNA but not in the corresponding primary tumor (Fig. 2b).

##### DNA methylation analysis in CTCs

*APC* and *SLFN11* promoter methylation was detected in genomic DNA (gDNA) derived from CTCs in 3/42 (7.1%) cases while *FOXA1* and *SHOX*2 promoters were detected in 2/42 (4.7%) and 1/42 (2.4%) patients, respectively. In contrast, *RASSFIA* promoter methylation was not detected in any of the gDNA derived from CTC samples. In total, at least one gene promoter was found methylated in 8/42 (19.0%) CTC fractions. In two patients two different gene promoters were methylated in the same sample. These patients had squamous cell carcinomas (SCC), were ex-smokers and one of them relapsed 15 months after surgery (Fig. 2b). The diagnostic specificity of real-time MSP assays for each gene was evaluated by analyzing the size-based enriched CTC fractions of 12 HD used as control group and revealed the absence of any methylated gene.

##### Methylation analysis in corresponding plasma cfDNA

All available corresponding plasma-cfDNA samples from these 42 NSCLC patients were analyzed. Promoter methylation was detected in 5/42 (11.9%) for *APC*, in 2/42 (4.7%) for *SHOXA* and in 4/42 (9.5%) for *SLFN11*. *RASSF1a* promoter was the most frequently methylated in plasma-cfDNA samples (6/42: 14.3%), whereas *FOXA1* promoter methylation was not detected in any sample (0/42: 0%). In total, at least one gene promoter was methylated in 14/42 (33.3%) plasma-cfDNA samples (Fig. 2b). Promoter methylation of *AP*C was observed in 2/12 (16.7%) HD whereas none of the HD plasma-cfDNA samples were methylated at the other tested promoters (0/12).

### Direct comparison of *APC, FOXA1, SHOX2, SLFN11* and *RASSFIA* promoter methylation in CTC, corresponding plasma-cfDNA and primary FFPEs tissues

A direct comparison of the methylation status of these five gene promoters in CTCs, corresponding plasma-cfDNA and paired FFPEs was performed. There was no any concordance between the DNA methylation status of CTC and plasma-cfDNA for any of the five studied genes (Additional file 1: Table S1). Moreover, there was no association between CTC fractions and 22 available paired primary tumors and the paired plasma-cfDNA regarding the promoter methylation status of any of the five studied genes (Additional file 1: Table S2).

### Clinical significance of *APC, FOXA1, SHOX2, SLFN11* and *RASSFIA* promoter methylation in early stage NSCLC

#### Training group

**Fig. 3:**
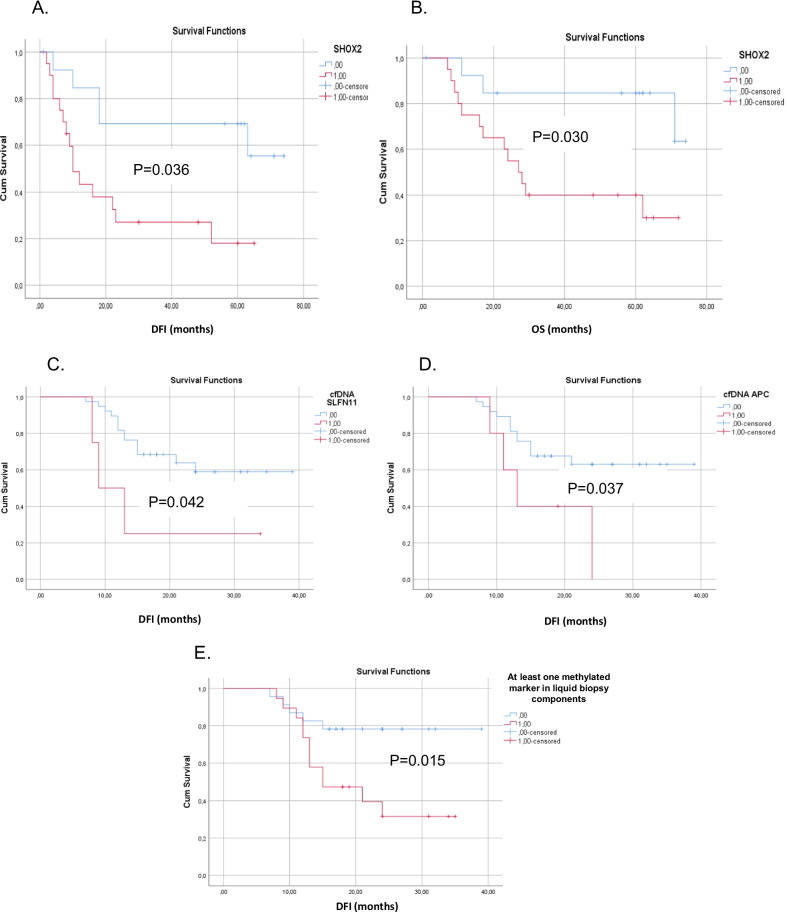
Kaplan–Meier estimates of **a** disease-free interval (DFI) in months for training group of tumor tissues of early NSCLC patients in respect to *SHOX2* promoter methylation status (*P* = 0.036), **b** overall survival (OS) in months for training group of tumor tissues of early NSCLC patients in respect to *SHOX2* promoter methylation status in tumor tissues (*P* = 0.030), **c** disease-free interval (DFI) in months for validation group of early NSCLC patients in respect to *SLFN11* promoter methylation in plasma-cfDNA (*P* = 0.042), **d** Disease-free interval (DFI) in months for validation group of early NSCLC patients in respect to *APC* promoter methylation in plasma-cfDNA (*P* = 0.037), **e** DFI in months for validation group of early stage NSCLC patients, in respect to the methylation of at least one gene in plasma-cfDNA or size-based CTC (*P* = 0.015)

During the follow-up period (73 months), 3 out of 35 patients without disease relapse died from other reasons and were thus not included in the survival analysis. In the remaining group, 19/32 (59.4%) patients relapsed and 16/32 (50%) died from the disease, (median follow-up: 39 months; (range 1–74 months). Kaplan–Meier analysis indicated that *SHOX2* methylation was significantly correlated with worse DFI (*P* = 0.036, log-rank test) and OS (*P* = 0.030, log-rank test) (Fig. 3a, b), while *APC, FOXA1, SHOX2, SLFN11* and *RASSFIA* methylation were not associated with clinical outcome.

#### Validation group

Kaplan–Meier estimates of the cumulative DFI of *SLFN11* or *APC* promoter methylation for NSCLC patients in plasma cfDNA were significantly different in favor of patients with non-methylated (*P* = 0.048 and *P* = 0.037, log-rank test, respectively, Fig. 3c, d). It is worth mentioning that the incidence of relapses was higher when at least one gene promoter was methylated in CTC or plasma-cfDNA in respect to patients where gene promoter methylation was not detected (Fig. 3e).

## Discussion

The current study describes for the first time the prognostic significance of DNA promoter methylation of a panel of five selected tumor suppressor genes in early stage NSCLC. Our analysis was performed firstly in a training group of primary NSCLC fresh-frozen tumor tissues and secondly in a validation group consisting of liquid biopsy components (size-based enriched CTC fractions and corresponding plasma-cfDNA) and the paired primary NSCLC tumor (FFPEs).

The selection of these five genes was based on their involvement in major hallmarks that lead to the development of cancer and on our meta-analysis of RNA-seq data from TCGA. To the best of our knowledge this is the first time that DNA methylation of these five genes is studied in CTCs of early stage NSCLC patients. Moreover, only a few studies have reported so far on the methylation status of these genes in plasma-cfDNA of early NSCLC patients. Detection of *RASSF1a* and *FOXA1* promoter methylation in plasma-cfDNA was a specific indication for the early detection of NSCLC [41]. Detection of *APC* and *RASSF1a* promoter methylation independently predicted disease-specific mortality in lung cancer patients [41]; these results are in line with our finding regarding the prognostic value of *APC* methylation in plasma-cfDNA. Detection of *SHOX2* methylation in serum has been shown to be a predictive marker for early stage NSCLC in combination with traditional markers [42].

According to our results, varying frequencies of promoter methylation has been found for these five genes. Our results clearly indicate that all tested genes are highly methylated in NSCLC tumor tissues, indicating that loss of expression of these tumor suppressor genes by methylation mechanisms is an early event in NSCLC tumorigenesis. *SLFN11* and *APC* were frequently methylated at high levels in both cancerous and adjacent tissues suggesting that hypermethylation of these genes may be associated with some environmental factors, such as chronic smoking as previously mentioned [43]. Moreover, there were some cases where methylation was detected in the adjacent tissues but not in the matched tumor tissues suggesting that these methylation changes were preneoplastic.

Our results indicated that in CTC and plasma-cfDNA all these five genes were methylated at significantly lower percentages than in primary tissues. It is worth mentioning that in plasma the number of samples found positive for almost all genes (except *FOXA1*) were higher than in CTC as the release of methylated DNA from apoptotic cells and necrotic cells seems to be significantly higher than DNA found in CTCs. This is also a possible explanation for the discrepancies detected between CTCs and plasma-ctDNA.

According to our findings, there was no concordance between the size-based enriched CTCs and paired primary tumors in respect to the promoter methylation status of any of the genes studied. A lack of concordance was also found for the same DNA methylation markers between plasma-cfDNA and matched primary tumors. A possible explanation for this finding could be based on tumor heterogeneity and rapid evolution through time, indicating that LB is reflecting the actual phase of tumor evolution [44, 45].

Τhe methylation of *SHOX2* promoter in primary tissues is associated with unfavorable prognosis in early stage NSCLC patients and the methylation of *SLFN11* and *APC* promoter in cfDNA is associated with decreased DFI. There was no any association between the methylation status of *RASSF1*a and *FOXA1* in CTCs, ctDNA and primary tumors. Finally Kaplan–Meier analysis revealed that the incidence of relapses was higher when at least one gene promoter was methylated in size-based CTC fractions or plasma-cfDNA in respect to patients where gene promoters were not methylated.

In conclusion, our findings indicate that the combination of DNA methylation analysis of tumor suppressor genes in CTCs and matched plasma-cfDNA provides significant prognostic information in patients with early stage NSCLC.

## Supplementary Information

**Additional file 1**. Comparison between SLFN11, SHOX2, FOXA1, RASSFIA and APC gene promoter methylation in CTC and corresponding paired plasma-cfDNA samples in early stage NSCLC (n = 42).

## Data Availability

All data generated or analyzed during this study are included in this published article (and its Additional files).

## Abbreviations

CTCs: Circulating tumor cells
ctDNA: Circulating tumor DNA
LB: Liquid biopsy
miRNAs: MicroRNAs
ncRNAs: Non-coding RNAs
TCGA: The CancerGenome Atlas
NSCLC: Non-Small-Cell Lung Cancer
SCC: Squamous Cell Carcinoma
ADC: AdenomaCarcinoma
HD: Healthy Donors
gDNA: Genomic DNA
FFPEs: Formalin-fixed paraffin-embedded tissues
SB: Sodium bisulfate
WGA: Whole genome amplification
ACTB: β-Actin
DFI: Disease free interval
OS: Overall survival
