# Supplementary material for: DNA methylation analysis of tumor suppressor genes in liquid biopsy components of early stage NSCLC: a promising tool for early detection
Source: Clin Epigenetics. 2022 May 10;14:61. doi: 10.1186/s13148-022-01283-x (PMC9092693; doi:10.1186/s13148-022-01283-x)
Supplement: Supplementary file 1 — Additional file 1. Comparison between SLFN11, SHOX2, FOXA1, RASSFIA and APC gene promoter methylation in CTC and corresponding paired plasma-cfDNA samples in early stage NSCLC (n = 42). [file 13148_2022_1283_MOESM1_ESM.doc]

**Suppl.Table 1:** Comparison between *SLFN11, SHOX2, FOXA1, RASSFIA* and *APC* gene promoter methylation in CTC and corresponding paired plasma-cfDNA samples in early stage NSCLC (n = 42)

|  | ***SLFN11*** | | | |
| --- | --- | --- | --- | --- |
|  |  | **Size-based CTC enriched fraction** | | |
| **Plasma-cfDNA** |  | Non-methylated | | Methylated |
|  | Non-methylated | 36 | | 2 |
|  | Methylated | 3 | | 1 |
|  |  | Concordance rate: 88.1% (P=0.265, k=0.222) | | |
|  | ***SHOX2*** | | | |
|  |  | **Size-based CTC enriched fraction** | | |
| **Plasma-cfDNA** |  | Non-methylated | | Methylated |
|  | Non-methylated | 39 | | 1 |
|  | Methylated | 2 | | 0 |
|  |  | Concordance rate: 92.8% (P=0.952, k=-0.33) | | |
|  | ***FOXA1*** | | | |
|  |  | **Size-based CTC enriched fraction** | | |
| **Plasma-cfDNA** |  | Non-methylated | | Methylated |
|  | Non-methylated | 40 | | 2 |
|  | Methylated | 0 | | 0 |
|  |  | Concordance rate: 95.2% (P=NA, k=NA) | | |
|  | ***RASSFIA*** | | | |
|  |  | **Size-based CTC enriched fraction** | | |
| **Plasma-cfDNA** |  | Non-methylated | | Methylated |
|  | Non-methylated | 36 | | 0 |
|  | Methylated | 6 | | 0 |
|  |  | Concordance rate: 80.1% (P=NA, k=NA) | | |
|  | **APC** | | | |
|  |  | **Size-based CTC enriched fraction** | | |
| **Plasma-cfDNA** | Non-methylated | 34 | 3 | |
|  | Methylated | 5 | 0 | |
|  |  | Concordance rate: 80.9% (P=0.677, k=-0.098) | | |

**Suppl.Table 2:** Comparison of *SLFN11, SHOX2, FOXA1, RASSFIA* and *APC* gene promoter methylation in primary tissues (FFPEs, n = 22) and a) size-based CTC-enriched fractions and b) plasma-cfDNA samples in early stage NSCLC

|  | ***SLFN11*** | | | | | |
| --- | --- | --- | --- | --- | --- | --- |
| **Primary FFPEs tissues** |  | **Plasma-cfDNA** | | | **Size-based CTC enriched fraction** | |
|  |  | Non-methylated | | Methylated | Non-methylated | Methylated |
|  | Non-methylated | 16 | | 2 | 18 | 0 |
|  | Methylated | 4 | | 0 | 4 | 0 |
|  | Concordance rate (P-value) | 72.7%(P=0.662) | | | 81,8%(P=NA) | |
|  | ***SHOX2*** | | | | | |
| **Primary FFPEs tissues** |  | **Plasma-cfDNA** | | | **Size-based CTC enriched fraction** | |
|  |  | Non-methylated | | Methylated | Non-methylated | Methylated |
|  | Non-methylated | 16 | | 2 | 18 | 0 |
|  | Methylated | 4 | | 0 | 4 | 0 |
|  | Concordance rate (P-value) | 72.7%(P=0.662) | | | 81,8%(P=NA) | |
|  | ***FOXA1*** | | | | | |
| **Primary FFPEs tissues** |  | **Plasma-cfDNA** | | | **Size-based CTC enriched fraction** | |
|  |  | Non-methylated | | Methylated | Non-methylated | Methylated |
|  | Non-methylated | 18 | | 0 | 17 | 1 |
|  | Methylated | 4 | | 0 | 4 | 0 |
|  | Concordance rate (P-value) | 81,8%(P=NA) | | | 77.3%(P=0.818) | |
|  | ***RASSFIA*** | | | | | |
| **Primary FFPEs tissues** |  | **Plasma-cfDNA** | | | **Size-based CTC enriched fraction** | |
|  |  | Non-methylated | | Methylated | Non-methylated | Methylated |
|  | Non-methylated | 16 | | 4 | 19 | 0 |
|  | Methylated | 2 | | 0 | 2 | 0 |
|  | Concordance rate (P-value) | 72.4% (P=0.662) | | | 86.4% (P=NA) | |
|  | **APC** | | | | | |
| **Primary FFPEs tissues** |  | **Plasma-cfDNA** | | | **Size-based CTC enriched fraction** | |
|  |  | Non-methylated | Methylated | | Non-methylated | Methylated |
|  | Non-methylated | 13 | 2 | | 15 | 0 |
|  | Methylated | 5 | 2 | | 7 | 0 |
|  | Concordance rate (P-value) | 68.2% (P=0.378) | | | 68.2% (P=NA) | |

**Suppl. Table 3**: Univariate and multivariate analysis (in the independent group of patients)

| **DFI** | | |
| --- | --- | --- |
| **UNIVARIATE ANALYSIS** | | |
| Age (>70 vs ≤70) | 2.070 (0.642-6.685) | P=0.223 |
| Histology (Non-Adeno vs Adeno) | 0.622 (0.344-1.123) | P=0.115 |
| Size (>5cm vs ≤5cm) | 1.778 (0.574-5.514) | P=0.319 |
| Smoking (yes or no) | 1.277 (0.522-3.127) | P=0.592 |
| **methylation of APC in cfDNA (yes or no)** | **4.344 (1.239-15.233)** | **P=0.022** |
| methylation of RASSF1A in cfDNA (yes or no) | 1.508 (0.399-5.785) | P=0.549 |
| **methylation of SLFN11 in cfDNA (yes or no)** | **5.017 (1.016-23.724)** | **P=0.042** |
| methylation of SHOX2 in cfDNA (yes or no) | 0.000 | P=0.989 |
| **MULTIVARIATE ANALYSIS** | | |
| **methylation of APC in cfDNA (yes or no)** | **3.631 (1.013-13.026)** | **P=0.048** |
| **methylation of SLFN11 in cfDNA (yes or no)** | **3.354 (1.160-9.704)** | **P=0.026** |
